# Rapidly Progressive Kidney Disease in a Patient With IgA Nephropathy and Focal Segmental Glomerulosclerosis Due to Nonadherence and Loss to Follow-Up

**DOI:** 10.7759/cureus.88734

**Published:** 2025-07-25

**Authors:** Zaheeruddin Sayed, Victoria Bellot, Javier J Mendoza Mulen

**Affiliations:** 1 Internal Medicine/Nephrology, Victory Medical Care PC, New York, USA

**Keywords:** end-stage renal disease, focal segmental glomerulosclerosis, iga nephropathty, immunosuppressive therapy, lost to follow-up, nonadherence, targeted-release budesonide

## Abstract

We report a case of a 63-year-old Hispanic female with hypertension and prediabetes, diagnosed with IgA nephropathy (IgAN) and focal segmental glomerulosclerosis (FSGS) in 2023. Despite detailed counseling about immunosuppressive therapy, the patient initially refused treatment due to fear of medication side effects. Over two years without consistent nephrology follow-up, her condition rapidly progressed to nephrotic syndrome and end-stage renal disease (ESRD). Later, immunosuppressive therapy, including corticosteroids, mycophenolate mofetil (MMF), and targeted-release budesonide (Tarpeyo), was initiated, but her kidney function continued to worsen. This case underscores the importance of ongoing nephrology follow-up and patient re-engagement in managing progressive glomerular diseases, especially in patients who initially decline therapy.

## Introduction

IgA nephropathy (IgAN) is the most common primary glomerulonephritis worldwide, with a range of clinical courses from stable kidney function to rapid progression to end-stage renal disease (ESRD) [1]. Coexisting focal segmental glomerulosclerosis (FSGS) lesions are present in a subset of patients and are associated with more aggressive disease and accelerated decline [2]. The Oxford classification is a validated histopathologic tool that helps stratify risk and guide clinical decision-making in IgAN [2]. Early initiation of supportive and immunosuppressive therapy, including blood pressure control, RAAS blockade, and corticosteroids, has been shown to benefit select patients with high-risk features [3]. However, patient refusal and inconsistent follow-up can delay intervention and result in poor outcomes. This case illustrates the rapid progression to ESRD in a patient with IgAN and FSGS who declined initial therapy and was lost to nephrology follow-up.

## Case presentation

A 63-year-old Hispanic woman with hypertension, hypothyroidism, and prediabetes initially presented in mid-2023 with bilateral lower extremity edema. At that time, she was under the care of another nephrologist. Initial laboratory evaluation revealed impaired kidney function and significant proteinuria. A kidney biopsy demonstrated IgA nephropathy (Oxford M1 E1 S1 T1 C1) with 30-40% interstitial fibrosis and tubular atrophy. Immunofluorescence showed 3+ mesangial IgA and 1+ C3 deposits. She was counseled regarding immunosuppressive therapy but declined treatment due to fear of medication side effects. Her decision was based on personal concerns and not influenced by cultural or socioeconomic barriers.

Over the following two years, she was lost to nephrology follow-up. In January 2025, she presented to our clinic with progressive generalized edema and additional complaints of heavy legs, dry mouth, and swelling in dependent areas. While awaiting laboratory results, she developed worsening shortness of breath, urinary incontinence, and increased edema, prompting evaluation in the emergency department. She was diagnosed and treated for uremic pericarditis. A repeat kidney biopsy during that admission revealed IgAN (Oxford M0 E0 S1 Tx Cx) with features of FSGS (cellular variant approaching tip), patchy interstitial fibrosis and tubular atrophy, and mild chronic interstitial inflammation. The biopsy specimen was insufficient for complete evaluation.

At the time of re-presentation, her blood pressure was markedly elevated, with readings of 186/98 mmHg (left arm) and 188/96 mmHg (right arm). Her home antihypertensive regimen included olmesartan 40 mg, metoprolol succinate 50 mg, and clonidine 0.1 mg daily. Other outpatient medications included levothyroxine, sitagliptin, ferrous gluconate, magnesium, vitamin C, and atorvastatin. Following hospitalization, her medication regimen was expanded to include nifedipine 60 mg daily and torsemide 20 mg BID. Immunosuppressive therapy was initiated with mycophenolate mofetil 250 mg BID and prednisone, and later transitioned to targeted-release budesonide (Tarpeyo) after a second opinion. Farxiga (dapagliflozin 10 mg), Drisdol (ergocalciferol), and Nephrovite were also added. Her blood pressure subsequently improved to 119/83 mmHg (left arm) and 128/88 mmHg (right arm), with a heart rate of 98 beats per minute and a weight reduction from 150 pounds to 131 pounds.

Throughout 2024 and 2025, laboratory data showed persistent nephrotic-range proteinuria (urine albumin-to-creatinine ratio (UACR) >11,000 mg/g at its peak), declining estimated glomerular filtration rate (eGFR) (as low as 11 mL/min/1.73 m²), and hypoalbuminemia, consistent with worsening glomerular damage and the progression of nephrotic syndrome. Anemia worsened over time, while parathyroid hormone (PTH) levels rose, indicating progression of chronic kidney disease. Notably, urinary red blood cells increased markedly by April 2025 (21-50/HPF), suggesting ongoing glomerular inflammation despite immunosuppressive therapy. Sequential laboratory results are summarized in Table 1.

**Table 1:** Sequential Laboratory Findings in Patient With IgA Nephropathy and FSGS Bold values exceed reference limits. eGFR: Estimated glomerular filtration rate (mL/min/1.73 m²), UACR: urine albumin-to-creatinine ratio (mg/g), PTH: parathyroid hormone, Uncalc.: uncalculated; value could not be computed due to exceeding the assay’s measurable range or limitations in available data, N/A: not available; data were either not collected or not reported at the respective time point. eGFR values in February 2023 and December 2023 (shown as ~46 and ~42, respectively) were calculated using a race-free version of the CKD-EPI creatinine equation.

Parameter	Reference Range (Units)	Feb 2023	Dec 2023	Dec 2024	Jan 2025	Feb 2025	Mar 2025	Apr 2025
Creatinine	0.5–1.0 (mg/dL)	1.3	1.4	2.7	3.2	4.3	3.4	2.9
eGFR	≥60 (mL/min/1.73 m²)	~46	~42	16	16	11	18	18
UACR	≤30 (mg/g)	N/A	2,900	3,450	4,400	11,432	6,063	Uncal.:
Albumin	3.5–5.2 (g/dL)	N/A	N/A	2.9	2.4	2.9	3.2	3.1
Hemoglobin	11.9–15.5 (g/dL)	N/A	N/A	11.9	11.7	13.7	10.1	9.3
Phosphorus	2.5–4.5 (mg/dL)	N/A	N/A	3.9	4.2	4.7	3.4	3.6
PTH	15–65 (pg/mL)	N/A	N/A	155	183	287	105	118
Total cholesterol	≤200 (mg/dL)	N/A	N/A	195	204	N/A	248	N/A
Triglycerides	≤150 (mg/dL)	N/A	N/A	140	257	N/A	212	N/A
Serum glucose	70–99 (mg/dL)	N/A	N/A	98	101	395	73	111
Urine glucose	Negative	N/A	N/A	N/A	>1,000	>1,000	1,000	>1,000
Dipstick protein	Negative	N/A	N/A	3+	3+	3+	3+	3+
RBCs in urine	0–2 (/HPF)	N/A	N/A	6–10	6–10	3–5	6–10	21–50

Despite multiagent therapy, including immunosuppression and supportive measures, her renal function and proteinuria remained largely unchanged. She has since been listed for kidney transplantation and is preparing to initiate dialysis.

## Discussion

This case demonstrates an unusually rapid progression of IgAN to ESRD within two years. Although approximately 20-40% of patients with IgAN progress to ESRD over two decades, the timeline can vary significantly depending on histologic features and clinical risk factors [1,2].

Early immunosuppressive therapy may slow progression in appropriately selected patients [3]. However, such decisions are influenced by biopsy findings. Histologic subclassification systems such as the Oxford classification and Haas classification provide prognostic insights, although overlapping pathologies like FSGS can complicate interpretation and therapeutic response [2,4].

Differentiating among primary, secondary, and genetic forms of FSGS is clinically important, as treatment strategies and outcomes differ significantly [5]. The Oxford classification (MEST-C score) has been validated across diverse cohorts and aids in risk stratification [6].

The NEFIGAN trial demonstrated that targeted-release budesonide significantly reduced proteinuria in patients with IgAN [7]. Subsequent follow-up data from the NefIgArd trial indicated that these benefits were sustained in a subset of patients over time [8]. Remission of proteinuria has consistently been associated with improved long-term renal outcomes in IgAN [9]. However, incomplete remission despite therapy is not uncommon and is often associated with persistent proteinuria and chronic histologic damage such as interstitial fibrosis [10]

In our patient, persistent nephrotic-range proteinuria, hypoalbuminemia, and worsening renal function despite immunosuppressive treatment reflected ongoing glomerular injury. Laboratory trends showed increasing proteinuria (>11,000 mg/g), worsening anemia, and rising parathyroid hormone (PTH), consistent with chronic kidney disease (CKD) progression. The rise in hematuria by April 2025 also suggests active glomerular inflammation.

A major contributor to her poor outcome was delayed initiation of therapy due to patient refusal, followed by a prolonged period without nephrology care. Although cultural factors have been explored in the context of medical nonadherence among some Hispanic patients, our case did not reflect a cultural refusal but rather personal concerns about side effects. Still, this case reinforces the need for clear communication, shared decision-making, and re-engagement strategies when patients initially decline treatment.

Additionally, although hypertension was present, her blood pressure management history was unclear due to the long period without medical supervision. Given the well-established role of uncontrolled blood pressure in accelerating CKD progression [5], we acknowledge it may have contributed to disease worsening. Monitoring and optimizing blood pressure remains an essential component of IgAN and FSGS care.

## Conclusions

Rapid progression to ESRD can occur in IgAN complicated by FSGS, especially when initial treatment is declined and nephrology follow-up is lost. This case highlights the importance of early diagnosis, continuous monitoring, and revisiting treatment options as patient perspectives evolve. It also underscores the physician’s role in actively encouraging patients to attend follow-up visits and adhere to medical recommendations. By fostering trust and maintaining consistent communication, providers can help prevent irreversible complications and improve long-term outcomes.
